# Multi-Institutional Analysis of Early Glottic Cancer from 2000 to 2005

**DOI:** 10.1186/1748-717X-7-122

**Published:** 2012-08-01

**Authors:** Naoki Hirasawa, Yoshiyuki Itoh, Shinji Naganawa, Shunichi Ishihara, Kazunori Suzuki, Kazuyuki Koyama, Takayuki Murao, Akiko Asano, Yoshihito Nomoto, Yoshimi Horikawa, Masahiro Sasaoka, Yasunori Obata

**Affiliations:** 1 Department of Radiology, Nagoya University Graduate School of Medicine, 65 Tsurumai-cho, Shouwa-ku, Nagoya, Aichi, 466-8550, Japan; 2 Department of Radiology, Hamamatsu University School of Medicine, 1-20-1 Handayama, higashi-ku, Hamamatsu, Shizuoka, 431-3192, Japan; 3 Department of Radiology, Tajimi Prefectural Hospital, 5-161 Maebata-cho, Tajimi, Gifu, 507-8522, Japan; 4 Department of Radiology, Ichinomiya Municipal Hospital, 2-2-22 Bunkyo, Ichinomiya, Aichi, 491-8558, Japan; 5 Department of Radiology, Toyohashi Municipal Hospital, 50 Hachiken Nishi, Aotake-cho, Toyohashi, Aichi, 441-8570, Japan; 6 Department of Radiology, Mie University School of Medicine, 2-174 Edobashi, Tsu, Mie, 514-8507, Japan; 7 Department of Radiology, Kainan Hospital, 396 Minami-honden, Maegasu-cho, Yatomi, Aichi, 498-8502, Japan; 8 Department of Radiology, Ise Municipal General Hospital, 3038 Kusube-cho, Ise, Mie, 516-0014, Japan; 9 Department of Radiological Technology, Nagoya University Graduate School of Medicine, 1-1-20 Daikou-minami, Higashi-ku, Nagoya, Aichi, 461-8673, Japan

**Keywords:** Radiotherapy, Glottic cancer, Chemoradiotherapy, Multiple institutes, Outcomes

## Abstract

**Background:**

The purpose of this study is to analyze the outcome of patients with early glottic cancer (GC) treated with radiotherapy (RT) with or without chemotherapy at 10 institutions in the Tokai District, Japan.

**Methods:**

Ten institutions combined data from 279 patients with T1-T2 GC treated with RT with or without chemotherapy between 2000 and 2005. The overall survival rate, disease-specific survival rate, and local control rate were evaluated in 270 patients, except for incomplete cases due to issues such as discontinuation, using the method of Kaplan-Meier and compared using the log-rank test. Results were considered statistically significant at the level of *p <* 0.05.

**Results:**

For 122 patients, the tumors were classified as T1a, while 64 patients had T1b tumors, and 84 patients had T2 tumors. In three cases of T1 tumors, the subtype was unknown. Combined chemoradiotherapy (CRT) was administered during each stage, and various chemotherapy drugs and regimens were used. The median follow-up period was 55.4 months. The 5-year LC rates for T1a, Tb, and T2 tumors in all patients were 87.9%, 82.7%, and 74.1%, respectively. The difference between T1a and T2 was statistically significant (p = 0.016). The 5-year LC rates for T1a, Tb, and T2 with CRT were 92.7%, 78.6%, and 80.7%, respectively, while the rates with radiation alone were 86.5%, 83.8%, and 64.4%, respectively. The difference between CRT and RT alone was not statistically significant in each stage.

**Conclusions:**

In this survey, CRT was performed for early GC at most institutions in clinical practice. Our data showed no statistical difference in the LC rates between CRT and RT alone in each stage. However, there was a tendency for the LCRs of the CRT group to be more favorable than those of the RT group in the T2-stage.

## Background

Many clinical studies in the 1990s showed that concurrent chemoradiotherapy (CRT) improved therapeutic efficacy for local advanced head and neck cancers more than radiotherapy (RT) alone did [1-4]. For example, in a phase III study [5] of patients with locally advanced laryngeal cancer, the rate of laryngeal preservation was 84% among patients receiving RT with concurrent cisplatin. These studies had great impact on the treatment of head and neck cancers in clinical practice. In evidence-based medicine (EBM), CRT is recommended for laryngeal preservation in advanced cases.

On the other hand, in early glottic cancer (GC), RT alone has been reported to be about 80-90% effective [6-15] for local control (LC) of T1 GC and 65-80% [6,7,10,13] effective for T2 GC. Some reports have shown that the LC for bulky T1 is only about 70% [14,15]. The LC rate with treatment by RT alone for bulky T1 and T2 is insufficient. According to recently published guidelines [16,17] for the treatment of head and neck cancer, all patients with T1-T2 laryngeal cancer should be treated, at least initially, with the intent of larynx preservation. The recommended strategies for early GC with the intent of larynx preservation are radiation therapy, transoral laser therapy, and partial laryngectomy. CRT is not recommended for early GC. However, most otolaryngologists in Japan believe that LC by RT alone is insufficient in early stage GC [18-20]. Reports recently released by several institutes in Japan [21-26] have indicated that chemoradiation for T2GC is promising and yields LC rates higher than those for RT alone. Therefore, chemoradiation for bulky T1 and T2 GC is used to improve LC in clinical practice in Japan. The details of CRT treatment for early GC, however, are unknown because no multicenter survey as a comprehensive study of the patterns of care in clinical practice has been conducted. The rates of chemoradiotherapy for early GC according to analyses of multiple institutions have been reported elsewhere [19]. In this study, we analyzed the treatment outcomes of patients with early GC treated with RT with or without chemotherapy at 10 institutions in the Tokai District, Japan.

## Methods

The Tokai Study group for Therapeutic Radiology and Oncology (TOSTRO) administered a questionnaire survey concerning radiotherapy for early GC in 2008. The questionnaire included questions about the stage of cancer (T1a, T1b, or T2 according to the 6th UICC International Union against the Cancer classification system, 2002), age, sex, performance status, histology, radiation dose, fraction size, method, total radiation dose, and combination chemotherapy among patients for whom RT was started between January 2000 and December 2005, at about the time when CRT became popular. The data from 10 institutions were recorded. This study was approved by the ethics committee of each participating hospital.

### Patient characteristics

Table 1 contains a summary of the clinical characteristics of the 270 patients who were included in the study. Among all patients, 260 (96%) were male, and 10 (4%) were female. According to the UICC system, for 122 patients, the tumors were classified as T1a, while 64 patients had T1b tumors. In only three cases of T1 tumors was the subtype unknown. The median ages of patients were 68 years (range 43–92) in the T1a group, 68 years (range 44–88) in the T1b group, and 66 years (range 46–83) in the T2 group. The patients included in the survey had a histologic diagnosis of infiltrative squamous cell carcinoma and no previous RT for head and neck neoplasms.

**Table 1:** Patient characteristics

	**T1a**	**T1b**	**T2**
Gender (n)			
Male	118	61	81
Female	4	3	3
Age (y)			
Median	68	68	66
Range	43–92	44–88	46–83
Performance status (ECOG)			
0–1	113	64	77
2	3	0	1
Unknown	6	0	6
Histology			
S.C.C.	122	64	84

Unknown subtype cases were taken off each table.

ECOG = Eastern Cooperative Oncology Group; S.C.C. = squamous cell carcinoma.

### Treatment

#### Radiotherapy

The various fractionation methods for each stage used during treatment of the 270 patients are summarized in Table 2. Conventional fractionation (CF) was used for 229 patients (84.8%), and hyperfractionation (HF) was used for 24 patients (8.9%). The other patients underwent accelerated hyperfractionation (AHF), CF plus HF, or CF plus AHF. CF was administered at 2 Gy per fraction per day. HF was delivered twice a day for a total dose of 67.6 Gy to 76.8 Gy, with 1.2 Gy to 1.3 Gy per fraction. In the T1a stage, CF was used for 96.7% of patients; in T1b, CF was used for 79.7%; and in T2, CF was for 71.4%. In the T2 stage, multiple fractionation was used more often than in the T1a stage. All patients were treated with parallel-opposed fields at 4 MV or 6 MV. Wedge filters of 15 or 30 degrees were used to optimize the dose distribution to achieve homogeneity of ± 5%. No prophylactic neck irradiation was performed in any of the cases.

**Table 2:** Fractionation method and radiation dose in each stage

	**T1a**	**T1b**	**T2**	**Total**
CF	118	51	60	229
median dose	68 Gy	68.2 Gy	70 Gy	70 Gy
(range)	( 60–74 Gy )	( 60–70.4 Gy )	( 64–82 Gy )	( 60–82 Gy)
HF	1	12	11	24
median dose	76.8 Gy	76.8 Gy	76.8 Gy	76.8 Gy
(range)	(76.8 Gy)	( 67.6-76.8 Gy )	(76.8 Gy )	(67.6-76.8 Gy)
AHF	0	0	5	5
median dose	-	-	72 Gy	72 Gy
(range)	( − )	( − )	(60–72 Gy)	(60–72 Gy)
CF + HF	2	0	4	6
median dose	68.8 Gy	-	67.1 Gy	67.1 Gy
(range)	(60.8-76.8 Gy)	( − )	( 54.4-76 Gy )	(54.4-76.8 Gy)
CF + AHF	1	1	4	6
median dose	68 Gy	67 Gy	70 Gy	68.5 Gy
(range)	( 68 Gy )	( 67 Gy )	( 56.5-71 Gy )	(56.5-71 Gy)
Total	122	64	84	270

CF = conventional fractionation ; HF = fractionation ; AHF = accelerated hyperfractionation.

#### Chemotherapy

The rate of combined CRT at each stage and the various chemotherapy drugs and regimens used are listed in Table 3. Of 92 patients who had undergone combination therapy, 81.5% had been treated with concurrent therapy, 9.8% with neoadjuvant therapy, 7.6% with alternative therapy, and 1.1% with adjuvant therapy. The most commonly used chemotherapeutic regimen was a high dose of CDDP/5-FU followed by UFT (tegaful-uracil) and weekly carboplatin. The other agents used in the present study included low-dose CDDP, low-dose CDDP/ 5-FU, docetaxel, and TS-1(tegaful/ gimeracil/ oteracil). Table 3 also shows the rates of CRT, classified by each T stage. The difference between the combination rate for T1a or T1b and that for T2 was statistically significant (T1a vs. T2, p < 0.001; T1b vs.T2, p < 0.001).

**Table 3:** Combination rates of chemotherapy and these regimens

	**T1a**	**T1b**	**T2**
Combination rate (%)	23†	22††	60
Regimen (n)			
Low dose CDDP (daily)	0	1	6
Low dose CDDP/5FU (daily)	0	1	2
High dose CDDP/5FU	9	5	17
Carboplatin (daily)	1	0	0
Carboplatin (weekly)	4	1	12
CDDP/5FU/DTX	0	0	5
DTX (Docetaxel)	1	0	2
UFT (oral antidrug)	12	5	6
TS-1 (oral antidrug)	1	1	0

† T1a vs. T2: p < 0.001; †† T1b vs. T2: p < 0.001.

### Statistical analysis

The median follow-up period was 55.4 months (range, 1.7 - 96 months). LC rates were assessed from the beginning of RT until evidence of recurrence or until laryngectomy. The overall survival rate, disease-specific survival rate and local control rate were evaluated in 270 patients, except for incomplete cases due to issues such as discontinuation, using the method of Kaplan-Meier, and compared using the log-rank test. Results were considered statistically significant at the level of *p <* 0.05.

## Results

### Local response, local control, and patterns of failure

Of 279 patients, six had finished with discontinuation or insufficient irradiation. Items from the six patients are described as follows. Two patients were interrupted for a simultaneous esophagus cancer. One case with 6 Gy was stopped by aggravation of an advanced esophagus cancer. The other completed the irradiation at 52 Gy, and surgery was performed for esophagus cancer. The 4 remaining patients interrupted for 18 Gy, 36 Gy, 52 Gy, and 53 Gy were changed into an operation, and each of these is controlled by operation. Evaluation of the primary tumor effect and a local control rate were analyzed except in these 6 patients and the 3 patients with T1 of an unknown subtype. For the reasons presented above, 270 of 279 patients were analyzed.

At the primary site, we observed 255 complete tumor responses (CRs) and 15 partial responses (PRs). The overall response rate (ORR) of the 270 patients was 100%. For 270 patients, the 5-year LC rates for T1a, Tb, and T2 were 87.9%, 82.7%, and 74.1%, respectively (Table 4).

**Table 4:** Local control rate and survival rate according to the staging

	**T1a**	**T1b**	**T2**	**p- value**
5-year local control rate	87.9%	82.7%	74.1%	T1a vs. T2 p = 0.016
				T1b vs. T2 p = 0.187
5-year overall survival	85.9%	90.3%	86.7%	N. S.
5-year cause-specific survival	100%	93.6%	93.5%	T1a vs. T1b p = 0.013
				T1a vs. T2 p = 0.006

N. S. = not significant.

**Figure 1:**
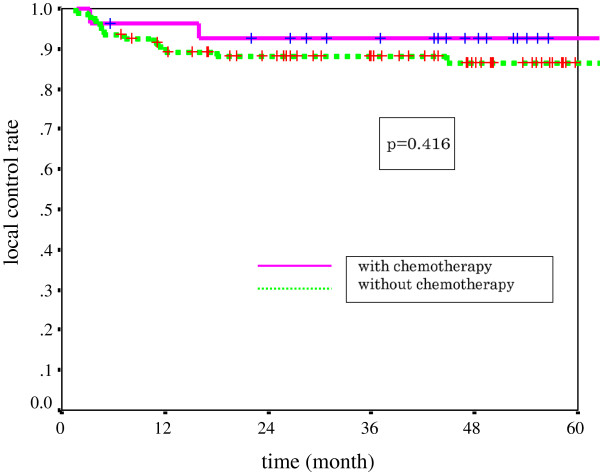
Local control rate for T1a tumor with or without chemotherapy.

**Figure 2:**
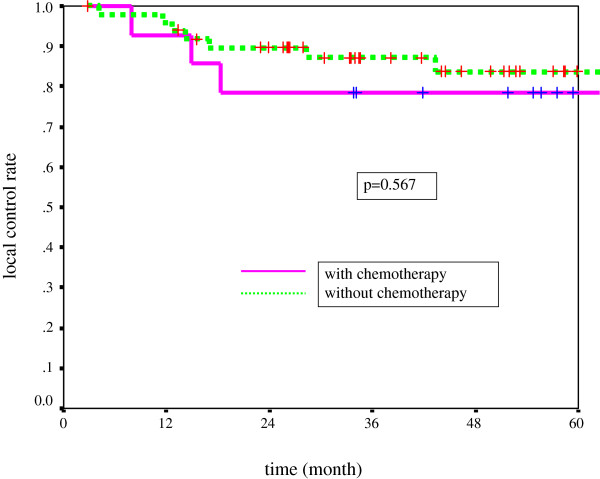
Local control rate for T1b tumor with or without chemotherapy.

**Figure 3:**
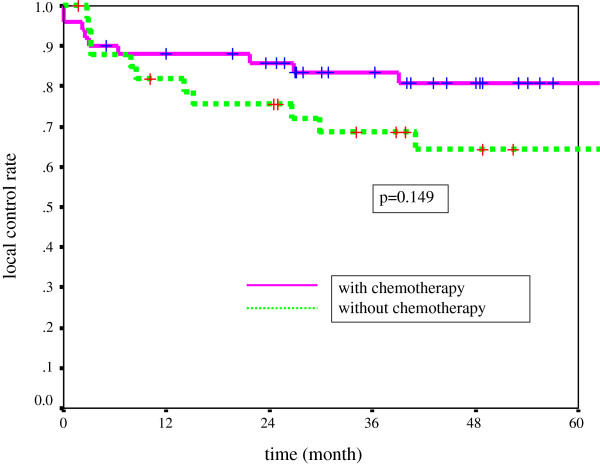
Local control rate for T2 tumor with or without chemotherapy.

The difference in the LC rate for T1a and that for T2 was statistically significant (p = 0.016). Of the 270 patients, 44 (including residual cases) developed recurrent disease at the primary site, and 2 had lymph node recurrences at the neck. Regarding treatment with or without chemotherapy, the 5-year LC rates for T1a, Tb, and T2 with CRT were 92.7%, 78.6%, and 80.7%, respectively, while the rates with radiation alone were 86.5%, 83.6%, and 64.4%, respectively (Table 5 and Figures 1, 2, and 3). The difference between CRT and RT alone was not statistically significant in each stage. However, there was a tendency for the LCRs of the CRT group to be more favorable than those of the RT group in the T2 stage.

**Table 5:** ***5-*year local control rate with or without chemotherapy**

	**With Chemotherapy**	**Radiation alone**	**p-value**
T1a	92.7%	86.5%	P = 0.416
T1b	78.6%	83.8%	P = 0.567
T2	80.7%	64.4%	P = 0.149

### Overall survival (OS) and cause-specific survival (CSS) rates

At the present time, 237 patients are living, and 33 have died. eight of the 33 died of GC. Twenty-six had intercurrent diseases, including 12 with a second primary cancer. The 5-year OS rates for T1a, Tb, and T2 were 85.9%, 90.3%, and 86.7%, respectively (Table 4). The difference between the sub-stage OS rates was not statistically significant. The 5-year CSS rates for T1a, Tb, and T2 were 100%, 93.6%, and 93.5%, respectively (Table 4).The difference between the CSS rate for T1a or T1b and that for the T2 stage was statistically significant (T1a vs. T2, p = 0.013; T1b vs. T2, p = 0.006).

### Complications

There were no severe acute complications with Grade 3 or above, and no patient required hospitalization due to late complications.

## Discussion

The survey showed that CRT was performed for early GC at most institutions in clinical practice in Japan. The rates of combination therapy for T1a, T1b, and T2 were 23%, 22%, and 60%, respectively (Table 3, T1a vs. T2, p < 0.001; T1b vs. T2, p < 0.001). The combination rate was higher in T2 in particular. However, CRT for early GC is not reported in well-known textbooks of radiation oncology [6,7]. Patients in the T2 stage with CRT underwent a high rate of therapy, and the 5-year LC of T2 GC was 80.7%. The outcome was favorable compared with that for radiation alone (64.4%), but there was no statistical difference between the outcomes in cases with chemotherapy and those without chemotherapy.

Recent studies have shown an improvement in LC for patients with T1 and T2 GC when total radiation is delivered following a high-dose fractionation [8,27] or hyperfractionation schedule [28-31] over a shorter overall treatment time. In the current study, 15% of the 270 patients were treated on hyperfractionation schedules. In the T2 stage, the tendency toward hyperfractionation was stronger than that in the T1a stage (Table 2). This selection of both CRT and hyperfractionation schedules demonstrates that most otolaryngologists and radiation oncologists in Japan are dissatisfied with the results of standard RT alone in early stage GC. As otolaryngologists have to perform salvage surgery for local failure, they tend to choose combined CRT to improve the LC rate [13]. In fact, many of the CRT regimens listed in Table 3 are used for advanced head and neck cancers. In short, we concluded that an improvement of CRT on LC of advanced head and neck cancers [1-5] had significant influence on the treatment of not only advanced cancers but also early GC in clinical practice in Japan. Several recent reports in Japan [21-26] have indicated that CRT for T2GC is promising and yields LC rates higher than those for RT alone. By means of these questionnaire surveys, we have demonstrated that CRT for T1 and T2 GC is used with the intent to improve LC in clinical practice at most institutions.

In recent papers [32,33], concurrent systemic platinum-based chemotherapy was recommended, preferably weekly cisplatin (30 mg/m2), for patients with T2 tumors with impaired cord mobility. As suggested in these studies, the current recommended strategy for T2 GC is CRT, and CRT for early GC will likely become more common worldwide in the near future.

In this survey, CRT was administered for early GC in clinical practice at most of the participating institutions; however, whether chemotherapy is effective for patients with very early GC, such as T1a or T1b, and whether or not chemoradiation therapy for these very early cancers is an overtreatment remain controversial.

## Conclusions

By means of a questionnaire survey regarding RT for early GC, we demonstrated that combined CRT was administered for early GC in clinical practice at most of the participating institutions. It is unknown whether chemotherapy should be combined with definitive radiotherapy for early GC. The results of this study showed no statistically significant difference in LCR between the RT group and the CRT group. However, there was a tendency for the LCRs of the CRT group to be more favorable than those of the RT group in the T2 stage. The results of this study with several limitations suggest that chemoradiation therapy might improve the local control rates of patients with T2-stage cancer.

## Competing interests

The authors declare that they have no competing interests.

## Authors’ contributions

NH and YI designed the study, acquired and interpreted the data, and wrote the manuscript. SN, SI, KS, KK, TM, AA, YN, YH, MS, and YO contributed to the study design and interpretation of data. SN and YO revised the manuscript. All authors have given final approval of the version to be published.

## Conflicts of interest statement

We declare no actual or potential conflict of interest.
